# Research on the Interaction of Genetic Algorithm in Assisted Composition

**DOI:** 10.1155/2021/3137666

**Published:** 2021-11-22

**Authors:** Han Hu

**Affiliations:** School of Architecture and Art, Central South University, Changsha, Hunan 410006, China

## Abstract

Computer-aided composition is an attempt to use a formalized process to minimize human (or composer) involvement in the creation of music using a computer. Exploring the problem of computer-aided composition can enable us to understand and simulate the thinking mode of composers in the special process of music creation, which is an important application of artificial intelligence in the field of art. Feature extraction on the MIDI files has been introduced in this paper. Based on the genetic algorithm in this paper, a platform of the sampling coding method to optimize the character representation has solved the traditional algorithmic music composition study. Music directly from the pitch and duration can be derived from the characteristics, respectively, in the form of a one-hot encoding independently said. Failure to the rhythm of the characterization of the pitch and duration are problems that lead to the inability of compositional networks to learn musical styles better. Rhythm is the combination of pitch and time values according to certain rules. The rhythm of music affects the overall style of music. By associating the pitch and time value coding, the rhythm style of music can be preserved better so that the composition network can learn the style characteristics of music more easily.

## 1. Introduction

Music is a complex art activity, which can purify people's hearts, enlighten wisdom, and adjust emotions, and it gradually has become an indispensable part of our lives [1]. In order to meet people's increasing demand for music work, an increasing number of scientists and researchers are involved in the exploration of music creation mode. With the progress of society and scientific and technological forces, the expression forms of music are becoming increasingly diversified, and the combination of computer and music promotes the rapid development of the music creation field. In the past, people's working music required creators to have solid professional music theoretical knowledge such as harmony, orchestration, melody, and rhythm. With the rise of artificial intelligence, many scientists have tried to apply artificial intelligence technology to music creation and made breakthrough progress. Artificial intelligence composition greatly lowers the threshold of music creation, which makes nonprofessionals participate in music creation even if they do not know musical instruments. At the same time, it improves the efficiency of professional song creation and provides rich music materials for music creators. Artificial intelligence composition has a wide application prospect, and it has room for display in advertising, social interaction, entertainment, and other scenes [2].

There are relatively many researches on the application of artificial intelligence composition technology in Western music, while there are few researches on the automatic creation of Chinese folk music, which is still in its infancy in China at present. Chinese folk music has a long history of thousands of years, rich in music resources and various forms, which can be described as an ideal material library for artificial intelligence composition. In the future, according to the needs of music creation, we can analyze the characteristics of national music, dig and learn the laws of national music creation by using artificial intelligence technologies, such as deep learning and machine learning, and build an automatic composition system with Chinese national music characteristics, which has important practical significance for the development and promotion of Chinese national music culture [3].

## 2. Genetic Algorithm

### 2.1. Genetic Algorithm Generation

The intersecting interpenetrating and promoting of life sciences, engineering, and other sciences is a remarkable feature of the development of modern science and technology, and the vigorous development of genetic algorithm reflects this feature and the trend of scientific development. Genetic algorithm is a kind of randomized search algorithm that draws lessons from natural selection and natural genetic mechanism in biology, and it is a computational model that simulates the biological evolution process of Darwin's genetic selection and natural elimination.

Genetic algorithm covers three main research fields: the research of basic genetic algorithm; genetic algorithm is used to optimize; and machine learning with classification system. Its main features are simplicity, versatility, strong robustness, suitability for parallel distributed processing, and wide application range [4].

### 2.2. Standard Genetic Algorithm (SGA)

Holland's genetic algorithm is often called Standard Genetic Algorithm (SGA), and the operation object of SGA is a group of binary strings (called chromosomes and individuals), that is, populations; here, each chromosome corresponds to the solution of a problem. Starting from the initial population, the selection strategy based on fitness ratio is adopted to select individuals in the current population, and crossover and mutation operations are used to generate the next generation population. This evolves from generation to generation until the expected termination conditions are met [5].

The basic idea of SGA is to start from a population representing the potential solution set of the problem, and a population is composed of a certain number of individuals encoded by genes. Each individual is an entity with characteristics called chromosomes. Chromosome is the main carrier of genetic material, that is, the collection of multiple genes, which determines the external performance of individual traits. Therefore, at the beginning, it is necessary to realize the mapping from phenotype to genotype, that is, coding. After the initial population comes into being, according to the principle of survival of the fittest and survival of the fittest, it evolves from generation to generation to produce better and better approximate solutions. In each generation, individuals are selected according to their fitness in the problem domain and combined, crossed, and mutated by means of genetic operators of natural genetics to produce a population representing a new solution set. This process will lead to the offspring of the population like natural evolution being more adaptable to the environment than the previous generation, and the optimal individual in the last population can be decoded as the approximate optimal solution of the problem [6].

There are three basic elements in the standard genetic algorithm:

1. Selection operation
2. Cross operation
3. Mutation operation

### 2.3. Characteristics of Genetic Algorithm

Genetic algorithm has strong robustness because it adopts many unique methods and techniques compared with ordinary optimization search methods. To sum up, there are mainly the following aspects:

1. The processing object of the genetic algorithm is not the parameter itself, but the encoded individual of the parameter set. This coding operation enables the genetic algorithm to directly operate structural objects, such as sets, sequences, matrices, trees, graphs, linked lists, etc., and even objects with three-dimensional structure.
2. Many traditional search methods are single-point search algorithms; that is, through some change rules, the solution of the problem moves from the current solution to another solution in the search space. On the contrary, the genetic algorithm adopts the method of dealing with multiple individuals in the population at the same time, that is, evaluating multiple solutions in the search space at the same time. More vividly, the genetic algorithm is to climb multiple mountains in parallel. This feature makes the genetic algorithm have better global search ability, reduces the risk of being limited to a local optimal solution, and is easy to parallelize.
3. In the standard genetic algorithm, the knowledge of the search space or other auxiliary information is basically not needed, but only the fitness function value is used to evaluate individuals, and genetic operations are carried out on this basis. It should be pointed out that the fitness function of the genetic algorithm is not only free from the constraint of continuous differentiability, but also its domain can be set arbitrarily.
4. Genetic algorithm does not use deterministic rules but uses probabilistic transition rules to guide its search direction.

**Figure 1:**
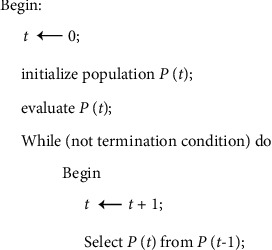
Process used by the genetic algorithm.

These characteristic techniques and methods make the genetic algorithm easy to use, strongly robust, and easy to parallelize; thus, it can be applied to a wider range of applications.  Figure 1 shows the process used by the genetic algorithm.

## 3. Network Structure Design of Melody Composition of Two-Part Folk Songs

Polyphony is a common form of folk songs; that is, it is composed of more than two independent melodies, among which two-part folk songs are the main form. When constructing the composition network, taking into account the multipart morphological characteristics of folk songs, therefore, based on the generation of one-part melody, this paper studies the characteristics of two-part folk songs; this paper puts forward a method based on curve fitting to create two-part folk song melody and designs an objective evaluation criterion suitable for evaluating two-part folk song melody. Through comparative experiments, the criterion is used to select the most suitable curve fitting function for generating two-part folk song melody.

### 3.1. Data Preprocessing

Artificial intelligence composition should first extract and represent music data, which will have an important impact on the subsequent composition [7]. This paper mainly extracts features from MIDI files, which is a file format commonly used by musicians to create and record music information and contains comprehensive music feature information. Feature representation is to represent the extracted music features into the data form that can be learned by the neural network model. Some feature representation often adopts one-hot coding form, and the music features represented by one-hot coding are independent of each other, and a large number of sparse matrices will be produced by using one-hot coding to represent the music features. Therefore, this simple music feature representation method cannot effectively represent the relationship between music features, so this paper proposes a lifting the sampling coding method based on genetic algorithm to optimize the feature representation.

#### 3.1.1. MIDI Feature Extraction

There are two commonly used audio processing formats for music storage: wav format and MIDI format [8]. wav format is the most primitive audio format, which is easy to obtain on the Internet and contains a large amount of information. However, it takes a lot of time to process wav waveform audio, and the accuracy of wav signal processing results will be lost. In this paper, another audio format, MIDI format music, is used as the data set. MIDI file is small, efficient in processing, and almost contains the required music features.

MIDI music may contain multiple tracks. First, it is necessary to separate the tracks and extract the main melody. Because the data used in this paper is self-collected MIDI music, which only contains the main melody track, it can be directly used for feature extraction without track separation. The algorithm for extracting note and time value features from MIDI music is shown in Algorithm 1.

#### 3.1.2. Up-Down Sampling Coding

Rhythm in melody is an important element to express feelings. Rhythm combines musical pitch and duration according to certain rules. Rhythm type is of great significance to shaping music style. A note without rhythm is like a soul without it. Rhythm can organize scattered pitch sequences together to form music, showing the artistic charm of music. In the past, algorithmic composition, the pitch, and time value features extracted from the melody of music are regarded as independent training features, failing to characterize the rhythm relationship between pitch and time value characteristics. Therefore, this paper proposes an up-down sampling coding method. The so-called upsampling coding method is to upsample each pitch according to the time value of each note and then send it to the network training to obtain a prediction model. The composing pitch sequence generated by the prediction model is then downsampled and decoded according to the time value to obtain the pitch sequence and time value sequence, respectively. This feature representation is more suitable for neural network models to learn the deep relationship between pitch and duration in the sequence so that the generated music can better retain the rhythm style of the original music.

The attributes of notes include pitch and duration, and the duration of notes in music can be divided into two-quarter notes, four-quarter notes, eight-quarter notes, etc. [9]. As shown in Table 1, the duration of each note is a multiple relationship, which is encoded according to the multiple relationships of the duration of each note.

A melody note is obtained from the training set, and its pitch sequence and time sequence are extracted as [64, 67, 69, 67, 69, 64, 67, 69] and [0.25, 0.25, 0.25, 1, 0.25, 0.25, 0.25], respectively. The pitch sequence is represented by simplified notation numerals as [3, 5, 6, 5, 6, 3, 5, 6]. Defining 0.125 as the minimum time value unit, according to the time value of each note and the time value multiple relationship of the minimum time value unit, the upsampling sequence is obtained by upsampling each pitch: [64, 64, 67, 67, 69, 69, 67, 67, 69, 69, 69, 69, 69, 69, 69, 69, 64, 64, 64, 67, 67, 67, 69]. If the relationship between the pitch and time value of this melody note is illustrated by the waveform, Figure 1 can be obtained.

(1) $$\begin{array}{c} u_{1}=3\left[u\left(t\right)-u\left(t-0.25\right)\right],\\ u_{2}\left(t\right)=5\left[u\left(t-0.25\right)-u\left(t-0.25\right)\right],\\ u_{3\left(t\right)}=6\left[u\left(t-0.5\right)-u\left(t-0.75\right)\right],\\ u_{4}\left(t\right)=5\left[u\left(t-0.75\right)-u\left(u-1\right)\right],\\ u_{5}\left(t\right)=6\left[u\left(t-1\right)-u\left(u-2\right)\right],\\ u_{6}\left(t\right)=3\left[u\left(t-2\right)-u\left(t-2.25\right)\right],\\ u_{7}\left(t\right)=5\left[u\left(t-2.25\right)-u\left(t-2.5\right)\right],\\ u_{8}\left(t\right)=6\left[u\left(t-2.5\right)-u\left(t-2.75\right)\right],\\ X\left(t\right)=u_{1}\left(t\right)+u_{2}\left(t\right)+u_{3}\left(t\right)+u_{4}\left(t\right)+u_{5}\left(t\right)+u_{6}\left(t\right)+u_{7}\left(t\right)+u_{8}\left(t\right). \end{array}$$

**Figure 2:**
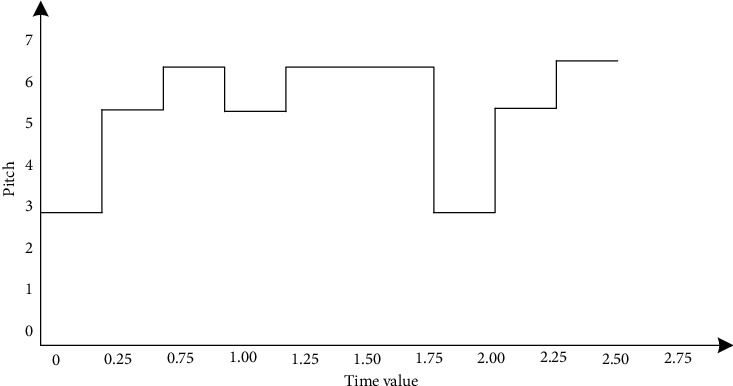
Relationship between pitch and duration.

**Figure 3:**
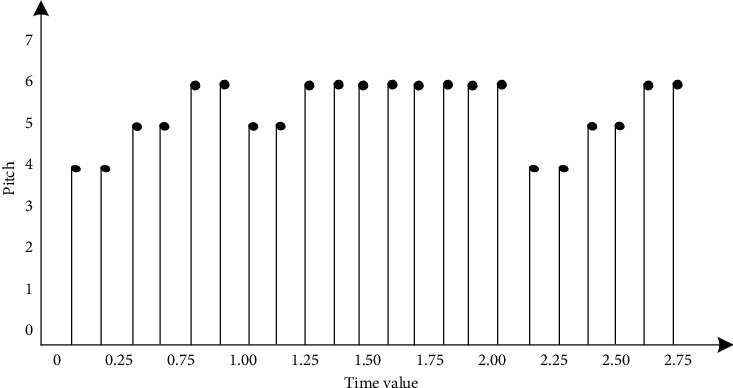
Sequence after sampling.

Figure 2 is represented by formula *X* (*t*) as follows. The continuous time signal *X* (*t*) is upsampled with the minimum value *τ* = 0.125, and the upsampled sequence is shown in Figure 3.

The continuous time signal *X* (*t*) is upsampled with the minimum value *τ* = 0.125, and the upsampled sequence is shown in Figure 3.

The upsampled sequence is expressed as follows:

(2) $$\begin{array}{c} X\left(t\right)|\left(t=nT\right)=X\left(nT\right)=R_{2}^{3}\left(n\right)+R_{2}^{5}\left(n\right)+R_{2}^{6}\left(n\right)+R_{2}^{5}\left(n\right)+R_{2}^{6}\left(n\right)+R_{2}^{5}\left(n\right)+R_{2}^{6}\left(n\right). \end{array}$$

The subscript *R*_*M*_^*N*^ (*n*) of the rectangular sequence (*n*) is extracted from formula (2) to obtain a sequence Q: [2, 2, 2, 2, 8, 2, 2, 2], which is downsampled and decoded by the time value coding table (Table 1) to obtain a downsampled time value sequence [0.25, 0.25, 0.25, 1, 0.25, 0.25].

**Figure 4:**
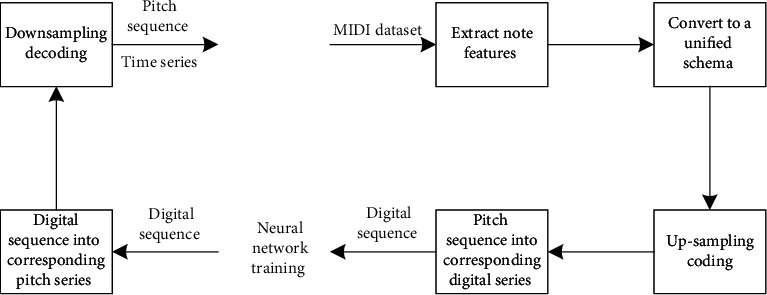
Up-down sampling coding process.

The up-down sampling coding process is described in detail with reference to Figure 4:

1. From MIDI training set, the music features such as mode, duration, and pitch of all music are extracted. The range of MIDI pitch in music is 0–127, and the duration is the relative duration of each note, and the unit is beat. If the quarter note is 1 beat, the duration is 0.5, which means half beat. The modes include all common modes in music and are represented by 0–23.
2. Combined with the mode conversion table stored in the database in advance, the MIDI music of different modes is converted into the pitch sequence and the corresponding time value sequence of the unified mode.
3. The pitch sequence is upsampled and encoded according to the corresponding time value sequence.
4. The upsampled sequence is normalized and transformed into a digital sequence suitable for neural network training.
5. The digital sequence of neural network training output is converted into MIDI composition pitch sequence output.
6. The output composing pitch sequence is downsampled and decoded to obtain the pitch sequence and the corresponding time value sequence.

### 3.2. Construction of the Composition Network

#### 3.2.1. Design of Composition Network Structure

Music is composed of many notes according to certain rules, and music generation can be understood as Seq-to-Seq sequence prediction problem [10]. Although traditional machine learning algorithms such as Gradient Boosting Decision Tree (GBDT), ensemble pruning algorithm and ensemble classification algorithm have been applied to various fields [11–13]. In this study, according to the feedback mechanism of cyclic neural network, which is good at solving the problem of note sequence prediction, this paper chooses Gated Recurrent Unit (GRU) [14], a variant of cyclic neural network, when constructing the composition network structure. GRU can not only deal with the problem of sequence generation, but also solve the problem of gradient disappearance and gradient explosion when cyclic neural network deals with long-distance sequence problems. Long Short-Term Memory (LSTM) is a temporal recursive neural network, which is suitable for processing and predicting important events with relatively long intervals and delays in time series [15]. LSTM already has many applications in technology [16]. When the prediction effect is equal, GRU needs less calculation, faster training, and less data to generalize when updating hidden states than LSTM. Musicians usually refer to the music melody before and after the current moment before the composing music. However, when using one-way GRU to build composition model, we often encounter a problem that we cannot learn from back to front. And Bidirectional Gated Recurrent Unit (Bi-GRU) can just solve this problem. Bi-GRU has proved effective in dealing with the correlation problem of long-time series, and it can capture the dependence between contextual music when used in music sequence generation. Therefore, considering the rules of music composition, the computing power of hardware, and the time cost, this paper adopts bidirectional cyclic neural network Bi-GRU when constructing the composition network.

**Figure 5:**
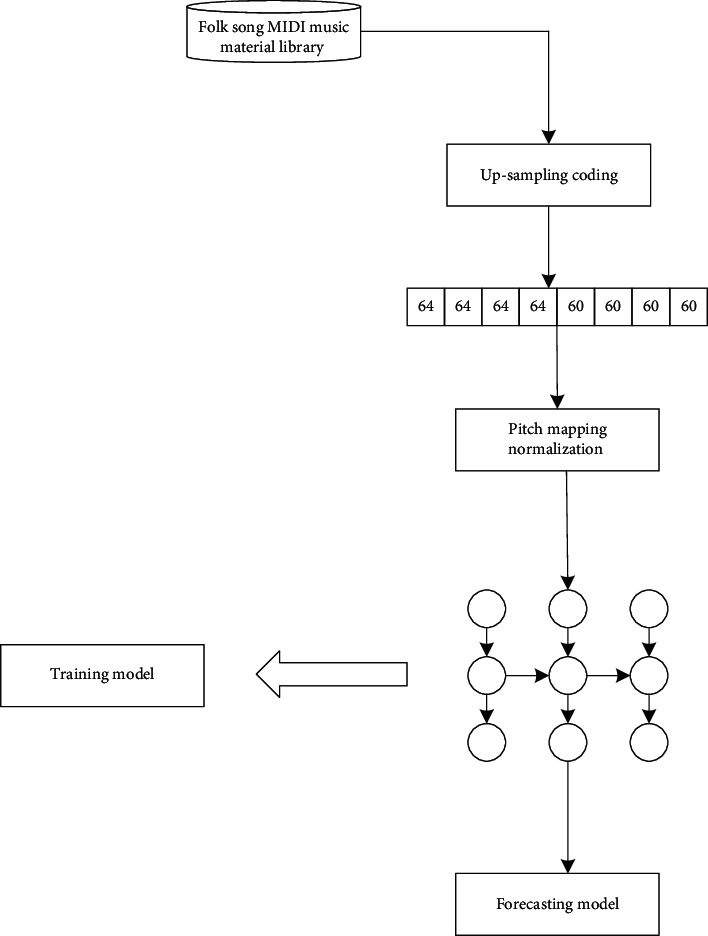
Training model of automatic composition network.

Figure 5 is an automatic composition network training model. The MIDI music in the MIDI material library of folk songs cannot be directly used for training, and the pretreated pitch sequence pitch and the corresponding time sequence durations need to be obtained after unified mode preprocessing. The pretreated pitch sequence and the corresponding time value sequence are, respectively, expressed as follows:

(3) $$\begin{array}{c} \text{pitches}=\left[{\text{pitch}}_{1},{\text{pitch}}_{2},\ldots ,{\text{pitch}}_{n}\right],\\ \text{durations}=\left[{\text{duration}}_{1},{\text{duration}}_{2},\ldots ,{\text{duration}}_{n}\right]. \end{array}$$

Firstly, the pitch sequence is preprocessed, and then the upsampled sequence, which is the input sequence of the network model, is obtained according to the upsampled coding of the sequence durations. The input sequence (upsampled sequence) *X* of the neural network is defined as follows:

(4) $$\begin{array}{c} X=\left[{\text{pitch}}_{1},\ldots ,{\text{pitch}}_{1},{\text{pitch}}_{2},\ldots ,{\text{pitch}}_{2},\ldots ,{\text{pitch}}_{e},\ldots ,{\text{pitch}}_{r}\right]. \end{array}$$

The pitch number of each pitch in the input sequence *X* after up- and downsampling is determined by the time value coding corresponding table of up- and downsampling.

According to the input sequence and network characteristics, formulate the corresponding network model training rules, by inputting the first *m* pitches of the input sequence, predicting the *m* + *l*th output pitch, the *m* + *l*th pitch of the predicted output is compared with the *m* + *l*th target pitch, calculate the error, update the network parameters, then shift the input pitch sequence backward by one pitch distance, and then predict the *m* + 2 note, compare the *m* + 2 note output by the network prediction with the *m* + 2 target note, and get the best prediction model with the smallest error after repeated iterative training.The expected output note set *Y* of the network model is represented as follows:

(5) $$\begin{array}{c} Y=\left[\begin{array}{c} {\text{pitch}}_{m+1}\\ {\text{pitch}}_{m+2}\\ {\text{pitch}}_{m+3}\\ \vdots \\ {\text{pitch}}_{n} \end{array}\right]. \end{array}$$

**Figure 6:**
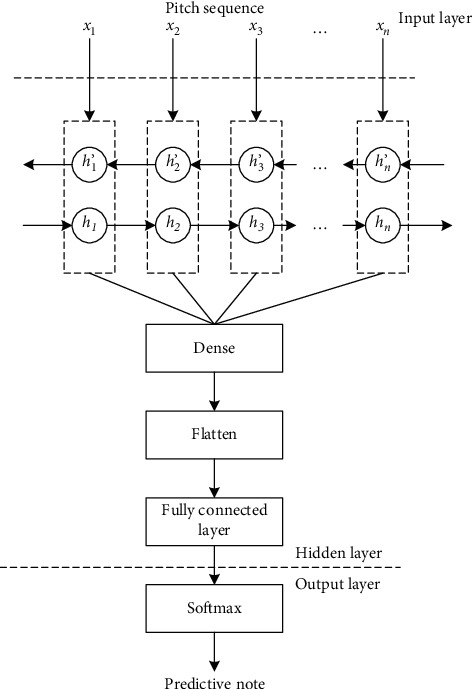
Network structure.

The network structure of the training model is shown in Figure 6. The network structure is divided into three parts, which are input layer, hidden layer, and output layer, from top to bottom.

The input layer is responsible for accepting input samples, assuming that the network accepts *Q* notes from the input sequence as an input sample. The input length of these *Q* pitches is called step size. Because of the large amount of training data, it is impossible to input the data into the network at one time, so the training data is divided into equal batches, and the training samples (i.e., batch_size) contained in each batch can be set by oneself. Different settings of batch_size will have a certain impact on the update of network weight and the convergence of the model. The correct choice of batch_size is to find the best balance between memory efficiency and memory capacity.

The hidden layer is composed of bidirectional cyclic neural network Bi-GRU, which includes two directions. The forward GRU can be used to learn the note feature information before the current time, and the backward GRU can be used to learn the note feature information after the current time. GRU output results in two directions are spliced as the output of the current time state. There are two hidden layers after GRU layer. The first hidden layer dense layer can reduce the dimension of features and improve the nonlinear ability of the first hidden layer. Dense layer can reduce the dimension of features and improve the nonlinear ability of the network model. The second hidden layer, flatten layer, flattens the features into one-dimensional vectors and then sends them to the full-connection layer. The generation of pitch sequence can be regarded as a multiclassification problem. Assuming that there are *F* possible pitch types, the full-connection layer maps the previously learned distributed features to these *F* target pitch vector values.

The output layer uses the softmax function to compress the vector values of target notes into intervals (0, 1) and can get the output probability of target notes, from which the maximum probability is selected as the predicted note output.

The process of neural network optimization is that the weight coefficients of the network are constantly updated so that the loss function value is minimized and the parameters converge. In this paper, the multiclassification cross entropy is used as the loss function to calculate the error between the predicted value and the real value. For each sample *i*, indicate *y*_*i*,*k*_ whether the sample belongs to category *K*. If the same category is 1, otherwise it is 0. Assuming that there are *K* label categories, and the probability that the *i*th sample is predicted to be the *K*th note category is *p*_*i*,*k*_, there are *M* samples in total, then the loss function LL of the data set is

(6) $$\begin{array}{c} LL=\frac{1}{M}\sum_{i}L_{i}=-\frac{1}{M}\sum_{i=1}^{M}\sum_{k=1}^{K}y_{i,k}\log\;p_{i,k}. \end{array}$$

When training neural network, the parameter optimization algorithm is of great significance to the convergence of the model. Choosing a learning algorithm suitable for training can greatly reduce training time and save computing resources. In this paper, Adam optimization algorithm is mainly used to adaptively update the learning rate of network parameters [17] so that the model has better performance on the training set. Compared with other optimization algorithms, Adam optimization algorithm has great advantages. It combines the advantages of adaptive gradient algorithm (AdaGrad) and root mean square propagation algorithm (RMSProp) and has better processing effect on large-scale data, which can improve the performance of sparse gradient and converge faster. Adam algorithm is one of the most popular optimization algorithms, which is widely used because of its good performance in practice. The calculation formula of Adam algorithm for network parameters is as follows:

(7) $$\begin{array}{c} u_{t}^{i}=\beta_{1}u_{t-1}^{i}+\left(1-\beta_{1}\right)g_{t},\\ u_{t}^{i}=\beta_{2}o_{t-1}^{i}+\left(1-\beta_{2}\right)g_{t}^{2},\\ {\hat{o}}_{t}^{i}=\frac{o_{t}^{i}}{1-\beta_{2}^{t}},\\ {\hat{u}}_{t}^{i}=\frac{u_{t}^{i}}{1-\beta_{u}^{t}},\\ △\theta_{t}^{i}=-\left(\frac{{\hat{u}}_{t}^{i}}{\sqrt{{\hat{o}}_{t}^{i}+\varepsilon }}\right)*\eta , \end{array}$$

where △*θ*_*t*_^*i*^ represents the increment of parameter *θ*_*t*_^*i*^ at time *t*, *g*_*t*_ is the gradient term of parameter *θ*_*t*_^*i*^ at time *t*, and *u*_*t*_^*i*^ is the first moment estimation of the gradient *g*_*t*_. *θ*_*t*_^*i*^ is a second-order moment estimate of the gradient *g*_*t*_. ${\hat{u}}_{t}^{i}$ and ${\hat{o}}_{t}^{i}$ are approximate unbiased estimates of *E*[*g*_*t*_] and *E*[*g*_*t*_^2^]. For the learning rate (e.g., 0.001), the default values of *β*_1_ and *β*_2_ are generally 0.9 and 0.999, respectively.

#### 3.2.2. Composition Flow Design

**Figure 7:**
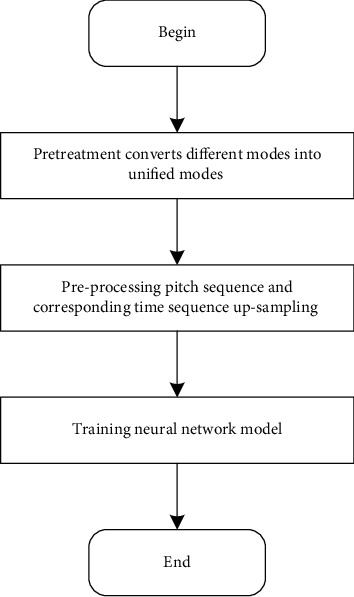
Training process.

**Figure 8:**
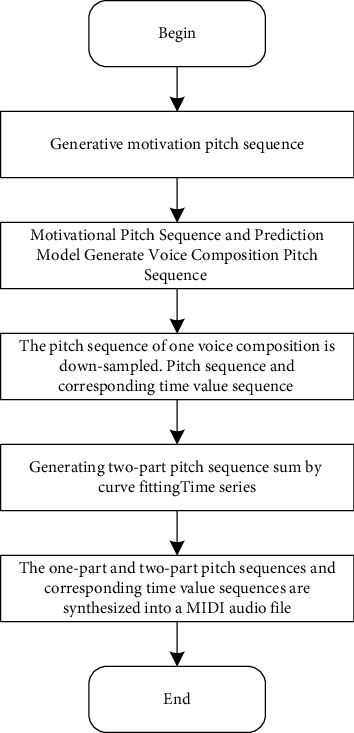
Composition process.

The algorithm composition process includes the training process and composition process, which are described in detail in Figures 7 and 8, respectively.

The training process steps are as follows:

1. All folk songs in MIDI format are stored in the music material library, and they contain folk songs with different modes. Firstly, the information of folk songs with different modes in the music material library is extracted, including music feature information, such as pitch, time value, and mode of music. According to the mode conversion table, the music with different modes is converted into the pretreatment pitch sequence and the pretreatment time value sequence of the unified mode.
2. Upsampling the preprocessed pitch sequence and the corresponding time sequence are upsampled to obtain the upsampled sequence as the input training set of the network model.
3. When the network model is trained, the neural network is trained by upsampling sequence, and the trained prediction model is obtained.

The composition process steps are as follows.

When a prediction model predicts and generates a pitch sequence, it first gives the model an initial input sequence and then generates a motivational pitch sequence according to the genetic algorithm as the initial input of the prediction model.

Combining the generated motive pitch sequence with the composition model, a voice composition pitch sequence is generated, and a voice pitch sequence and a corresponding time value sequence are obtained by downsampling and restoring the composition pitch sequence.

Combining one-part pitch sequence and the corresponding time sequence, two-part pitch sequence and time sequence are generated by the polynomial fitting method, and the pitch correction is carried out.

The pitch sequence and the time value sequence generated for the first part and the second part are converted into the corresponding MIDI audio format for output. You can set the speed, beat, timbre, and mode of MIDI music according to your needs. In the data preprocessing, we have converted the pitch sequence into a unified mode. Here, it can be converted into other modes and output again when MIDI is synthesized.

If a tune of *C* is converted to *D*, and if the tonic pitch base _note *D* of the mode *D* to be converted is known to be 62, the MIDI pitch sequence of *C* can be expressed by the set < *R*, *V* > according to the mode conversion table. *R* denotes simple spectral digital symbols, *V* denotes different octave values, and −1, 0, and 1 denote low octave, octave, and high octave, respectively. The scale interval relationship of each mode is full + full + half + full + full + half. It is expressed as scale = [ai] = [0, 2, 2, 1, 2, 2, 2, 1]. Each MIDI pitch sequence after transposition is obtained from the following formula:

(8) $$\begin{array}{c} {\text{Note}}_{D}={\text{base}}_{{\text{note}}_{C}}+V\times 12+\overset{R-1}{\underset{i=0}{\sum }}a_{i}. \end{array}$$

### 3.3. Subjective Evaluation

The quality evaluation of algorithmic composition has always been a difficult point, and a good quality evaluation index has important guiding significance for further improving the music quality generated by algorithmic composition. Music art belongs to a subjective perception of human beings. In this paper, fuzzy comprehensive evaluation and entropy weight method are used to evaluate the algorithmic composition.

The establishment steps of the music subjective evaluation model are as follows:

(1) Determine the evaluation index *U*, which is defined as *U* = {*u*1, *u*2, *u*3,…, *uk*}, where *k* represents the number of indexes. The evaluation index *U* = {please to the ear, innovation, hierarchy, harmony and style clarity}.
(2) Determine the grade of each index score, which is defined as *B* = {*b*1, *b*2,…, *bn*}, where *n* represents the number of grade grades. In this paper, the values of *B* are {1, 2, 3, 4}, which are respectively expressed as {bad, average, good, very good}.
(3) Determine the weight, which reflects the importance of each index and is defined as *a* = {*a*1, *a*2, *a*3,…, *ak*}. This paper uses the entropy weight method to objectively determine the weight of each evaluation index.
Firstly, the score of each evaluation index is normalized to between [0, 1], and the formula is as follows:
  (9) $$\begin{array}{c} y_{i,j}=\frac{b_{ij}-\min\left(b_{1j}b_{2j},\ldots ,b_{nj}\right)}{\max\left(b_{1j}b_{2j},\ldots ,b_{nj}\right)-\min\left(b_{1j}b_{2j},\ldots ,b_{nj}\right)}, \end{array}$$
where *b*_*ij*_ represents the score of the *i*th person under the *j*th index, and *y*_*i,j*_ represents the normalized value of the *i*th person score under the *j*th index.
Next, the information entropy *E*_*j*_ of each index is determined:
  (10) $$\begin{array}{c} h_{ij}=\frac{y_{ij}}{\sum_{i=1}^{n}y_{ij}}, \end{array}$$
  (11) $$\begin{array}{c} E_{j}=-\frac{\sum_{i=1}^{n}h_{ij}\text{In}h_{ij}}{\ln\left(n\right)\left(j=1,2,3,\ldots ,k\right)}. \end{array}$$
In formulas (10) and (11), when *h*_ij_ = 0, limit $\underset{h_{ij}⟶0}{\lim}h_{ij}\ln\;h_{ij}=0$.
Finally, the weight of each index *j* is calculated according to the value of each information entropy:
  (12) $$\begin{array}{c} W={\left\{W_{j}\right\}}_{l\times m}=\frac{1-E_{j}}{m-\sum_{j=1}^{m}E_{j}},\;j=1,2,3,\ldots ,m. \end{array}$$
(4) To determine the comprehensive score of music, firstly, the fuzzy comprehensive evaluation matrix *g* is obtained according to the following formula:
  (13) $$\begin{array}{c} Q=\left[\begin{array}{c} Q_{1}\\ Q_{2}\\ \ldots \\ Q_{k} \end{array}\right]=\left[\begin{array}{ccc} h_{11} & \cdots & h_{1n}\\ \vdots & \ddots & \vdots \\ h_{k1} & \cdots & h_{kn} \end{array}\right], \end{array}$$
Among them, *Q*_1_, *Q*_2_, Q_3_,…, *Q*_*k*_, respectively, represent the scoring situation of all scoring personnel of *k* indicators, and *h*_*kn*_ in *Q*_*k*_ = {*h*_*k*1_, *h*_*k*2_,…, *h*_*kn*_} indicates the proportion of all scoring personnel of grade *n* for this indicator *k*.

Finally, the fuzzy comprehensive evaluation matrix *Q* is multiplied by each index weight *W*, and the corresponding grade with the highest value in the result *S* is the final score of the music. The calculation formula is as follows:

(14) $$\begin{array}{c} S=WQ=\left[s1,s2,\ldots ,sn\right]. \end{array}$$

## 4. Experiment and Composition Evaluation

The data in this paper mainly comes from the MIDI folk song data set collected independently. After preprocessing the data set, it is sent to the network for training.

### 4.1. Network Training and Optimization

After the network is built, it is necessary to train the network. In the process of network model training, the initial parameters of the model need to be determined firstly, and the setting of different parameters has a great influence on the convergence of the network. The MIDI training set is upsampled, and the values of the upsampled sequence are normalized as inputs. And the pseudocode for the network model training algorithm is shown in Algorithm 2.

**Figure 9:**
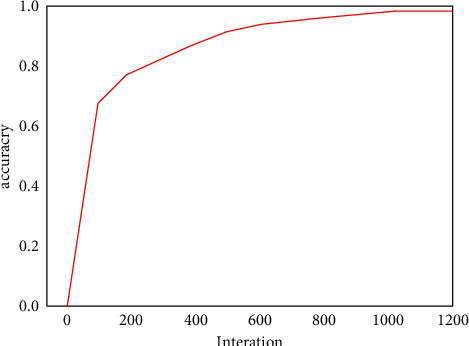
Influence of iteration times on training effect.

**Figure 10:**
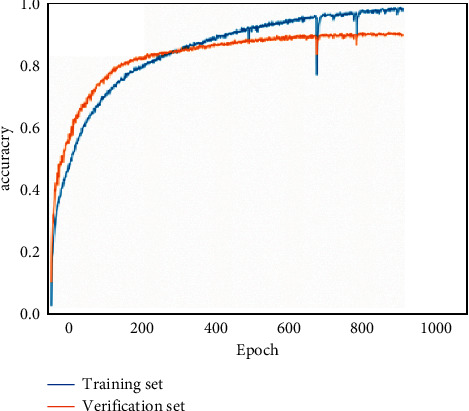
Accuracy change process of training set and verification set.

After many experiments, the final process of determining the network model parameters is described as follows:

(1) 200 folk songs were collected, and the number of pitches extracted after data preprocessing was 22454. These pitches need to be upsampled according to the corresponding time value and then sent to the network for training, and the total number of upsampled samples is 77394. This experiment adopts the way of cross-validation, and 20% of the data in the training set is divided as the test set; a verification set is also set in each round of training to update the network model parameters. When each iteration is completed, the parameter values of the training network model are saved. When the next iteration is completed, if the effect of this round of model is better than that of the previous round of model, the parameter values of the previous round of the network model are covered. Finally, the test set is used to verify the best the network model. Set the step size time_step = 100 for each round of input. The total number of notes is 21, so the number of neurons in the output layer is 21.
(2) The neural network model is constructed with the deep learning framework Keras, which has six layers. The hidden layer consists of three Bi-GRU layers: dense layer, flatten layer, and fully connected layer. In the first GRU layer, the time step and the number of the features in the output layer are defined. After GRU layer, a hidden-layer dense layer is added to reduce feature dimensions and improve the nonlinear ability of the model. Flatten layer is responsible for flattening the feature data and then transmitting it to the full-connection layer, which outputs the vector values of each note type. The softmax function compresses the output vector value of each note type into an interval (0, 1) and calculates the probability of output of each note type. The excessive number of neurons in each layer will make the network model complex, and the training will consume more memory resources. To improve the training speed as much as possible and reduce the loss of memory resources, the optimal model parameters are determined, and different hidden layer numbers and the number of units in each layer are set and experimented for many times. The experimental results are as follows:
  - When there is only one Bi-GRU layer in the hidden layer, the influence of different neuron numbers on the accuracy is shown in Table 2.
  - When there are two layers of Bi-GRU in the hidden layer, the first layer has 512 neurons, and the influence of different number of neurons in the second layer on the accuracy is shown in Table 3.
  - When there are three layers of Bi-GRU in the hidden layer, there are 512 neurons in the first layer and 512 neurons in the second layer. The influence of different neuron numbers in the third layer on the accuracy rate is shown in Table 4.
According to the above experimental results, when there are three Bi-GRU layers and the number of units in each Bi-GRU layer is 512, 512, and 512, the training effect is the best, and the accuracy of training set is 99.32%, while that of test set is 88.75%, as shown in Table 5.
(4) In the training process, Adam optimization algorithm is used to update each parameter value, and the error between the predicted value and the target value is calculated by cross entropy loss function. Set batch_size = 128; that is, the number of samples in one iteration is 128. Epoch = 1000; that is, all training samples have completed training iterations 1000 times. In order to find the best neural network model for note prediction, several groups of experiments with different iteration times were carried out, and the influence of iteration times on training effect is shown in Figure 9.
(5) In the process of training, when the data set is small, the model performs well on the data set; however, using the test set to test whether the prediction effect is too different is not ideal. This is called overfitting in deep learning. To avoid overfitting and improve the accuracy of the test set, dropout method is adopted in this experiment. Dropout temporarily discards some neuron nodes by setting overfitting probability *p*. During training, these neuron nodes will not work. In order to prevent overfitting, their weights will not be updated. After many iterative experiments, the overfitting probability *p* of the hidden layer is set to 0.8. The accuracy changes of the training set and verification set are shown in Figure 10.
(6) In the process of composing music, the trained prediction model will generate a pitch according to the input initial sequence every time, and the generated pitch sequence will be obtained after *N* iterations. At this time, the pitch sequence is still upsampling sequence, and the pitch sequence and the corresponding time value sequence need to be reduced by downsampling. Finally, set the beat and speed, and synthesize the pitch sequence and the corresponding time value sequence into MIDI audio format for output.

### 4.2. Method Analysis and Comparison

A single-layer feedback neural network is used to solve combinatorial optimization problems [18], this was a prototype of the first RNN, and then it was developed in [19], and it is also the simplest RNN model today that contains a single self-connected node. In order to validate the feasibility of this algorithm, three RNN-based algorithms proposed in the Magenta project of Google Lab, namely, basic_rnn, mono_rnn, and attention_rnn composing model, and the algorithm composing model designed in this paper are tested respectively, and the accuracy and loss rate of the models are compared. In order to ensure the objectivity of experimental comparison, the same MIDI data set collected independently in this paper is used for composing model experiments of different algorithms, and the iterations are 1000 times.

As shown in Table 6, it is the comparison result of the accuracy and loss rate between the three algorithm models of Magenta project and the composition model of this paper.

From the experimental results, we can see that the algorithm model designed in this paper has the highest accuracy and the smallest loss rate compared with the other three models. It can be seen that the algorithm model in this paper has a good performance on the training set in feature representation and network structure design and is suitable for learning the internal structure features of note sequences from the training set. In order to fully compare the music effects generated by each algorithm, this paper also compares the chromatographic vector distribution map of music predicted by different algorithm models.

The so-called chromaticity vector distribution map counts the pitch distribution in music; in order to obtain the chromaticity vector distribution, firstly, Fourier transform is carried out on the music to obtain a spectrogram, divide the spectrogram into different octaves, the spectrum is divided into 12 intervals according to logarithmic proportional intervals within an octave. According to the twelve average rate in music theory, the frequency between every two octave levels is 2 times, and the frequency between semitones increases in equal proportion of 2 (1/12), so each interval corresponds to one semitone, which corresponds to the note names in turn: C, C#, D, D#, E, E#, F, F#, G, G#, A, A#, B, and the abscissa of the chromatographic vector distribution map are named after these twelve sound names, and the corresponding semitones in different octave intervals are superimposed to finally form a 12-dimensional chromatographic vector. In order to further describe the difference of pitch distribution between different music, the chromatographic contrast index is defined:

(15) $$\begin{array}{c} V_{tr}=\frac{\sum_{j=1}^{12}v_{i}^{\prime }}{\sum_{j=1}^{12}v_{i}^{\prime }},\;v_{1}^{\prime }\geq v_{2}^{\prime }\geq \cdots \geq v_{12}^{\prime }. \end{array}$$

Chromatographic contrast can quantitatively compare the pitch distribution of different music. If the chromatographic contrast is higher, it reflects the fact that the pitch in music is more concentrated and the chromatographic contrast is lower, which shows that the pitch distribution in music is scattered. The pitch distribution of a music has a great relationship with the mode and scale adopted by the music.

**Figure 11:**
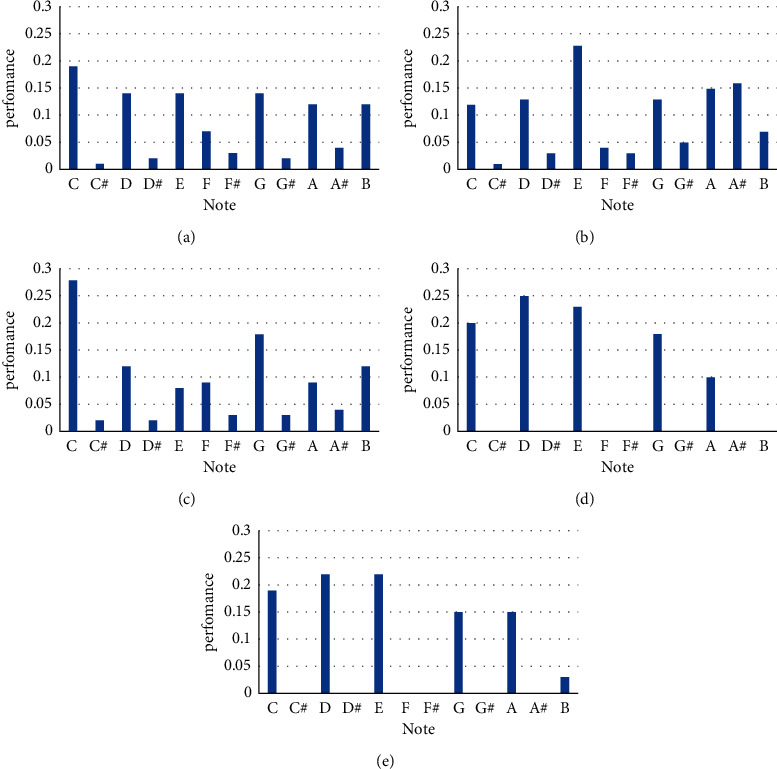
Comparison of generated music by different models. (a) Training sample: “only folk songs respect relatives.” (b) basic_rnn generated music. (c) mono_rnn generated music. (d) attention_rnn generated music. (e) This design model generates music.

Figure 11 shows the chromatographic vector distribution between the training sample music and the music generated by the model in this paper and the music generated by the three algorithms of Google Labs Magenta Project.

From the experimental results, the chromatographic vector distribution reflects the difference of pitch distribution among different generated music.  Figure 11(a) is the chromatographic vector distribution of the training sample. It can be seen that the components are evenly distributed on the pentatonic scale. Because Chinese traditional folk music mainly adopts the pentatonic scales of palace, quotient, angle, sign, and feather, which correspond to the sound names: C, D, E, G, and A in turn, the pitch distribution mainly focuses on these five components, and the vector distribution of music generated by the composition model designed in this paper is closest to that of the training samples, as shown in Figures 11(a)–11(e). And the music generated by the three algorithm models of Google Labs Magenta Project, the energy is evenly distributed to the 12 notes that constitute the twelve average laws, among which the scales in C major (C, D, E, F, G, A, and B) account for the most proportion. Therefore, the basic elements such as pitch, melody color, and interval of music created under different melodies will be different, and the melody will bring people different auditory feelings.

As shown in Table 7, by calculating the chromatographic contrast, it can be seen that when *K* = 5, the chromatographic contrast of music generated by the three algorithm models of Magenta Project in Google Lab is low, while the chromatographic contrast of music generated by the composition model and training samples in this paper is high. Chromatographic contrast can also distinguish folk music represented by the pentatonic scale from Western music represented by twelve average laws. To sum up, the composition model designed in this paper can learn the internal structural relationship of the training sample sequence well and is more suitable for generating music with typical national style.

## 5. Conclusion

This paper discusses the problem of computer-aided composition and deeply understands and simulates the composer's thinking mode in the special process of music creation, which is an important application of artificial intelligence in the art field. Feature extraction of MIDI file is carried out in this paper. A platform sampling coding method based on genetic algorithm is proposed to optimize the character representation, it solves the problem that the traditional algorithm can directly derive features from the pitch and duration of music in music composition learning and encode them in a heat-independent form, which fails to characterize the rhythm of pitch and duration, resulting in the composition network being unable to learn music style better. Rhythm is the combination of pitch and time value according to a certain rule. The rhythm of music affects the overall style of music. Through the correlation between pitch and time value coding, the rhythm style of music can be better preserved, and the composition network can learn the style characteristics of music more easily.

## Figures and Tables

**Algorithm 1:**
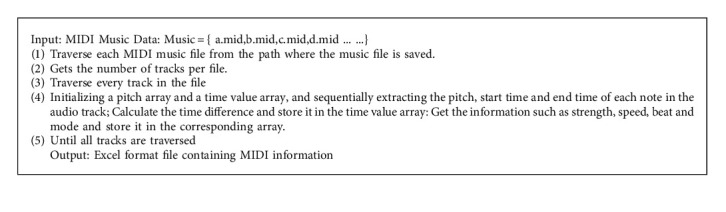
Algorithm for obtaining note and time value characteristics.

**Algorithm 2:**
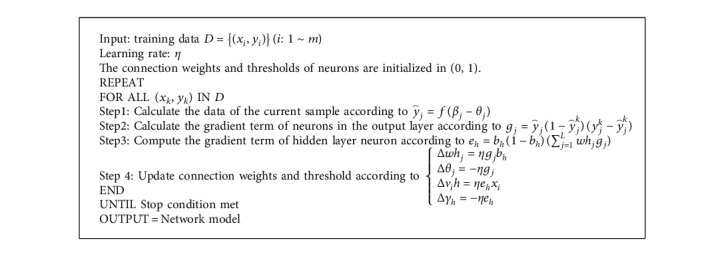
The pseudocode for the network model training algorithm.

**Table 1:** Correspondence table of note time value coding.

Note name	Time value	Code
Thirty-second note	0.125	1
Sixteenth note	0.25	2
Sixteen points with dots	0.375	3
An eighth note	0.5	4
Eight-point dot note	0.75	6
Quarter note	1	6
Quarterly dot note	1.5	12
Dichotomous note	2	16

**Table 2:** Influence of different numbers of neurons on the accuracy in a single hidden layer.

Number of neurons	Training set accuracy (%)	Test set accuracy (%)
128	83.68	79.68
256	85.67	82.30
512	96.89	82.77

**Table 3:** Influence of the number of undirected neurons on the accuracy when the second layer hides the layer.

Number of neurons	Training set accuracy (%)	Test set accuracy (%)
128	96.31	85.62
256	97.75	86.61
512	98.02	86.16

**Table 4:** Influence of different numbers of neurons on the accuracy in three hidden layers.

Number of neurons	Training set accuracy (%)	Test set accuracy (%)
128	95.86	87.15
256	98.31	88.73
512	99.32	88.75

**Table 5:** Number of hidden layers and neurons.

Number of hidden layers	Number of neurons
Layer 1: Bi-GRU layer	512
Second layer: Bi-GRU layer	512
Third layer: Bi-GRU layer	512

**Table 6:** Comparison of accuracy and loss rate of the algorithm model.

Network model name	Accuracy	Loss rate
basic_rnn	94%	0.15
mono_rnn	79%	0.64
attention_rnn	84%	0.55
Composition model of this article	99%	0.03

**Table 7:** Calculation results of chromatographic contrast.

Music title	Chromatographic contrast
Only folk songs respect relatives	1.0
basic_rnn generate music	0.79
mono_rnn to generate music	0.76
attention_rnn to generate music	0.71
This paper designs a model to generate music	0.97

## Data Availability

The experimental data used to support the findings of this study are available from the corresponding author upon request.
